# Supplementary material for: Analysis of isobaric quantitative proteomic data using TMT-Integrator and FragPipe computational platform
Source: Nat Commun. 2026 Mar 2;17:4010. doi: 10.1038/s41467-026-70118-7 (PMC13136390; doi:10.1038/s41467-026-70118-7)
Supplement: Supplementary file 1 — Supplementary Information [file 41467_2026_70118_MOESM1_ESM.pdf]

PSM

Spectrum	Phospho Site Localization	Purity
f01.19628.19628.2	DET(0.026)FGEY(0.260)S(0.679)DS(0.036)DEK	1
f01.29614.29614.4	EDLHPDPEIQS(0.831)VKDET(0.831)FGEY(0.829)S(0.412)DS(0.096)DEK	1
f01.31594.31594.4	EDLHPDPEIQS(0.659)VKDET(0.667)FGEY(0.210)S(0.410)DS(0.053)DEK	0.62
f01.31668.31668.5	EDLHPDPEIQS(0.263)VKDET(0.534)FGEY(0.534)S(0.625)DS(0.045)DEK	0.63
f01.31673.31673.3	EDLHPDPEIQS(0.697)VKDET(0.691)FGEY(0.243)S(0.308)DS(0.061)DEK	1
f02.29773.29773.4	EKEDLHPDPEIQS(0.928)VKDET(0.928)FGEY(0.056)S(0.048)DS(0.040)DEK	0.83
f02.31010.31010.4	EDLHPDPEIQS(0.922)VKDET(0.016)FGEY(0.024)S(0.020)DS(0.017)DEK	0.92
f02.31139.31139.5	EDLHPDPEIQS(0.130)VKDET(0.085)FGEY(0.759)S(0.014)DS(0.012)DEK	0.58
f02.31975.31975.4	EDLHPDPEIQS(0.634)VKDET(0.634)FGEY(0.634)S(0.064)DS(0.033)DEK	0.58
f02.32964.32964.4	EDLHPDPEIQS(0.185)VKDET(0.365)FGEY(0.705)S(0.681)DS(0.064)DEK	1

filter PSMs
- TMT/iTRAQ labeled
- check reference channel intensity & precursor ion purity
- MS/MS intensity check, best PSM selection
- remove contaminants

multi-site

<table>
<tr><th>Index</th><th>Peptide</th><th>Channel 1</th><th>Channel 2</th><th>Channel...</th></tr>
<tr><td>sp|O00533|NCHL1_HUMAN_1127_1139_1_1_S1127</td><td>EDLHPDPEIQsVKDETFGEYSDSDEK</td><td>R11</td><td>R12</td><td>...</td></tr>
<tr><td>sp|O00533|NCHL1_HUMAN_1127_1139_1_1_Y1136</td><td>EDLHPDPEIQSVKDETFGEySDSDEK</td><td>R21</td><td>R22</td><td>...</td></tr>
<tr><td>sp|O00533|NCHL1_HUMAN_1127_1139_2_0</td><td>EDLHPDPEIQSVKDETFGEYSDSDEK</td><td>R31</td><td>R32</td><td>...</td></tr>
<tr><td>sp|O00533|NCHL1_HUMAN_1127_1139_2_2_S1127T1132</td><td>EKEDLHPDPEIQsVKDEtFGEYSDSDEK</td><td>R41</td><td>R42</td><td>...</td></tr>
<tr><td>sp|O00533|NCHL1_HUMAN_1127_1139_3_3_S1127T1132Y1136</td><td>EDLHPDPEIQsVKDEtFGEySDSDEK</td><td>R51</td><td>R52</td><td>...</td></tr>
<tr><td>sp|O00533|NCHL1_HUMAN_1132_1139_1_0</td><td>DETFGEYSDSDEK</td><td>R61</td><td>R62</td><td>...</td></tr>
</table>

collapse to single-site
- keep only PSMs with localized sites
- use only PSMs with the single localized site when available
- use median of all localized if only found with other sites

single-site

<table>
<tr><th>Index</th><th>Peptide</th><th>Channel 1</th><th>Channel 2</th><th>Channel...</th></tr>
<tr><td>sp|O00533|NCHL1_HUMAN_S1127</td><td>EDLHPDPEIQsVKDETFGEYSDSDEK</td><td>R11</td><td>R12</td><td>...</td></tr>
<tr><td>sp|O00533|NCHL1_HUMAN_T1132</td><td>EKEDLHPDPEIQsVKDEtFGEYSDSDEK;EDLHPDPEIQsVKDEtFGEySDSDEK;</td><td>med(R41, R51)</td><td>med(R42, R52)</td><td>...</td></tr>
<tr><td>sp|O00533|NCHL1_HUMAN_Y1136</td><td>EDLHPDPEIQSVKDETFGEySDSDEK</td><td>R21</td><td>R22</td><td>...</td></tr>
</table>

Supplementary Figure 1. Examples illustrate the detailed process of generating single-site ratio tables, starting from the PSM list to multi-site ratios and finally to single-site ratios. Ratio values are denoted by colored text in the channel columns.

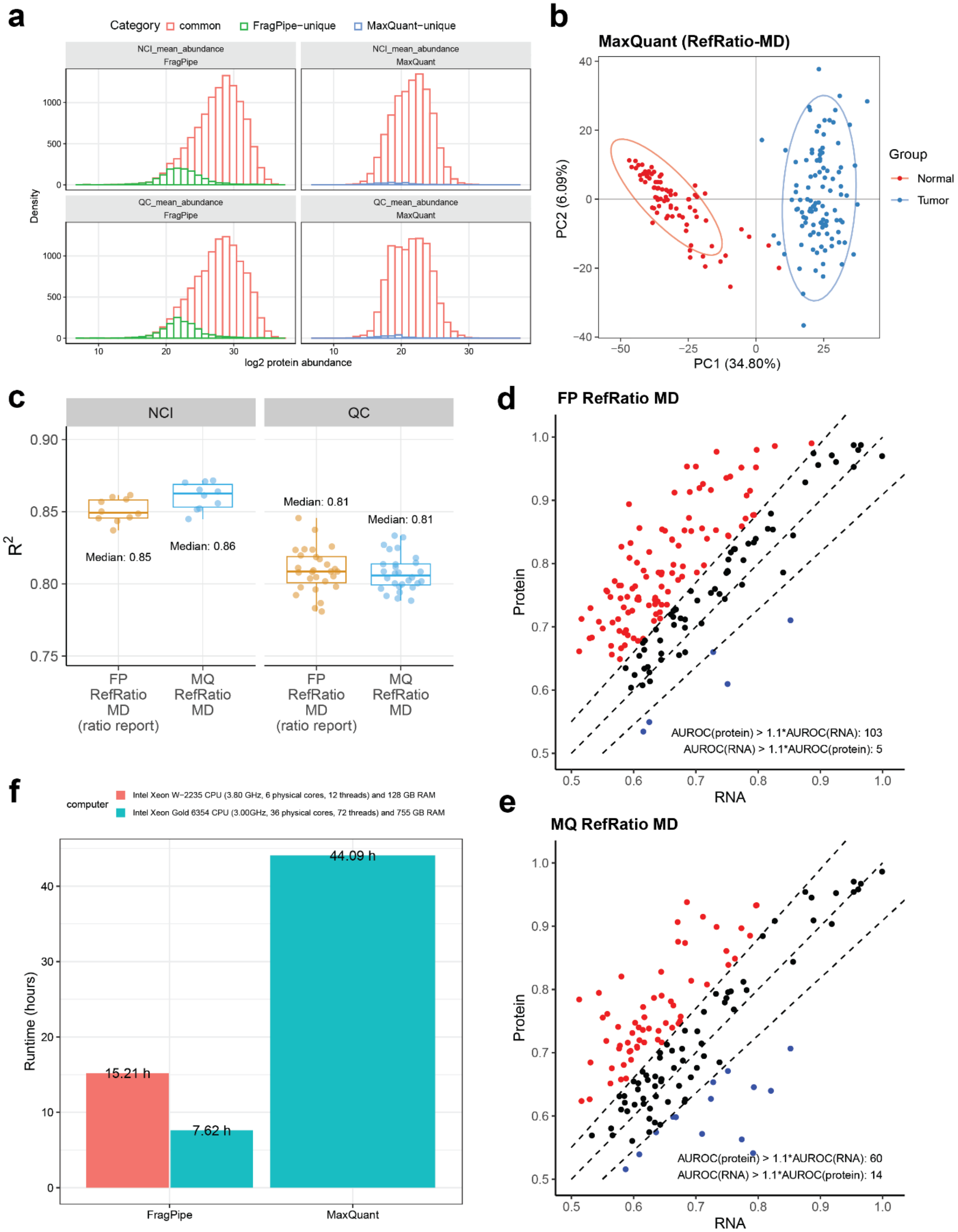
a
Category common FragPipe−unique MaxQuant−unique
NCI_mean_abundance FragPipe
NCI_mean_abundance MaxQuant
QC_mean_abundance FragPipe
QC_mean_abundance MaxQuant
Density
log2 protein abundance
b
MaxQuant (RefRatio-MD)
PC2 (6.09%)
PC1 (34.80%)
Group
Normal
Tumor
c
NCI
QC
R2
Median: 0.85
Median: 0.86
Median: 0.81
Median: 0.81
FP RefRatio MD (ratio report)
MQ RefRatio MD
FP RefRatio MD (ratio report)
MQ RefRatio MD
d
FP RefRatio MD
Protein
RNA
AUROC(protein) > 1.1*AUROC(RNA): 103
AUROC(RNA) > 1.1*AUROC(protein): 5
e
MQ RefRatio MD
Protein
RNA
AUROC(protein) > 1.1*AUROC(RNA): 60
AUROC(RNA) > 1.1*AUROC(protein): 14
f
computer
Intel Xeon W−2235 CPU (3.80 GHz, 6 physical cores, 12 threads) and 128 GB RAM
Intel Xeon Gold 6354 CPU (3.00GHz, 36 physical cores, 72 threads) and 755 GB RAM
Runtime (hours)
15.21 h
7.62 h
44.09 h
FragPipe
MaxQuant

Supplementary Figure 2. Performance evaluations using the ccRCC whole proteome dataset. (a) Average protein abundance distributions of NCI and QC samples quantified by FragPipe and MaxQuant. Red bars show 10660 proteins quantified by both tools, green bars show 1550 proteins unique to FragPipe, and blue bars show 151 proteins unique to MaxQuant. **(b)** PCA plot of MaxQuant median-centered protein quantification from 103 tumor and 79 normal samples. **(c)** Box plots showing the protein-level abundance correlation (R^2) between replicate runs for NCI and QC samples using ratio-to-reference data. The box in each plot captures the IQR with the bottom and top edges representing the Q1 and Q3, respectively. The median (Q2) is indicated by a horizontal line within the box. The whiskers extend to the minima and maxima within 1.5 times the IQR below Q1 or above Q3. R^2 values were calculated from all pairwise replicate comparisons among the five NCI samples (10 comparisons) and the eight QC samples. The median R^2 values are labeled for each method. FP represents FragPipe, MQ represents MaxQuant, RefRatio represents ratio-to-reference normalization with a real reference and MD represents the use of median-centering normalization. **(d)** Scatter plot comparing KEGG pathway membership predictions (AUROC values) between FragPipe protein data (RefRatio MD) and RNA data. Each dot represents a KEGG category; red dots indicate higher AUROC values from protein data, blue dots indicate higher AUROC values from RNA data, and black dots indicate no noticeable difference. **(e)** Same as (d) using protein data from MaxQuant (RefRatio MD). **(f)** Runtime of FragPipe with TMT-Integrator and MaxQuant for analysis of the ccRCC whole proteome dataset (23 plexes × 25 fractionations, 575 LC-MS files) on two platforms. The Windows desktop has 6 cores (12 threads) and 128 GB RAM, and the Linux server has 36 cores (72 threads) and 755 GB RAM. MaxQuant's runtime is reported only for the Linux server as running on the Windows desktop was substantially longer. Source data are provided as a Source Data file.

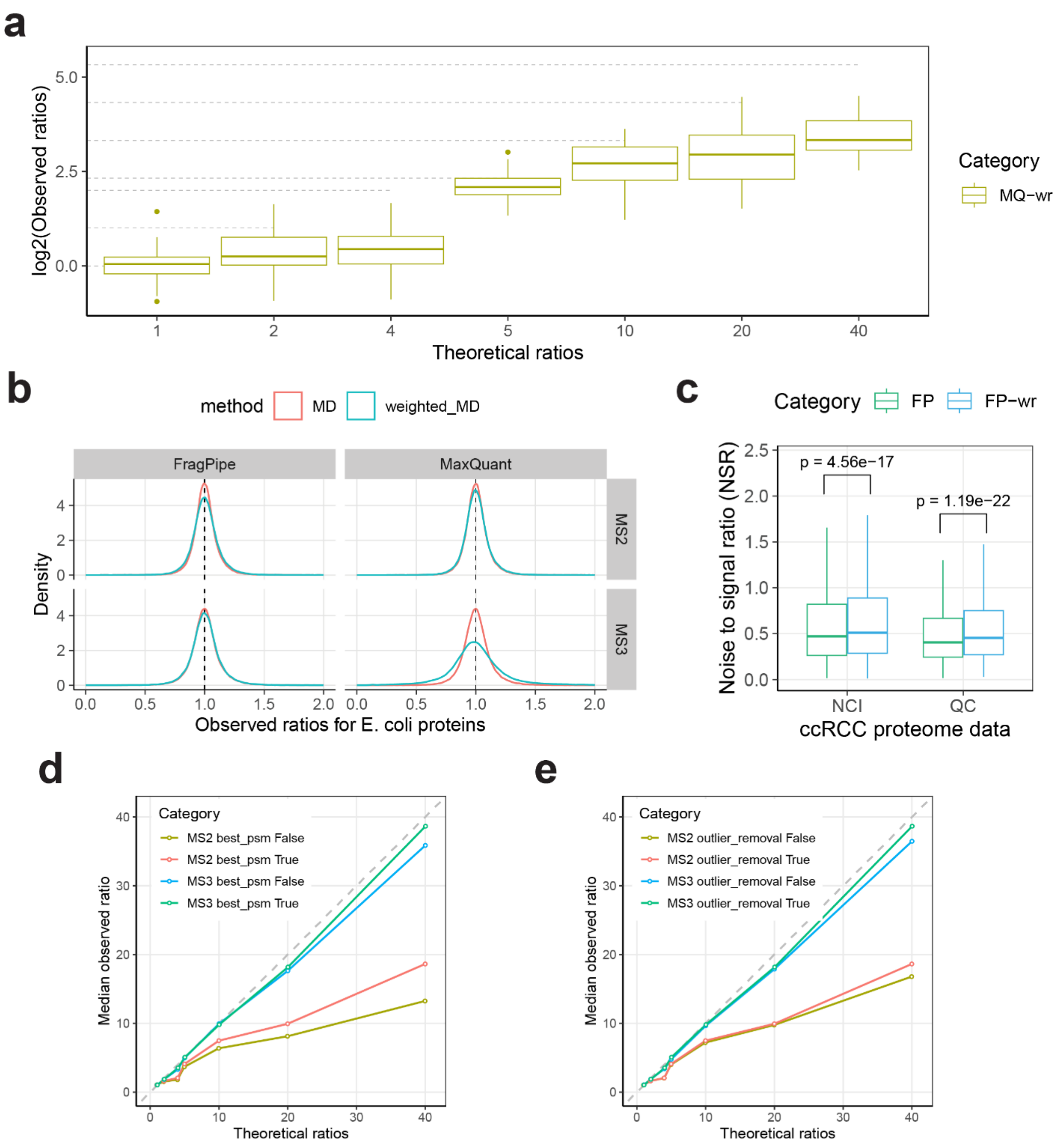

Supplementary Figure 3. Performance evaluations using the spike-in dataset. (a) Box plots showing the observed ratio distributions of 12 spike-in proteins compared to the theoretical ratios (grey dashed lines) in the MS3 data from the MaxQuant weighted median ratio method. The box in each plot captures the IQR with the bottom and top edges representing the Q1 and Q3, respectively. The median (Q2) is indicated by a horizontal line within the box. The whiskers extend to the minima and maxima within 1.5 times the IQR below Q1 or above Q3. **(b)** Comparison of two ratio aggregation methods (i.e., median ratio and weighted median ratio) based on the observed ratio distributions of the *E. coli* proteins reported by FragPipe and MaxQuant, respectively. Line colors represent different ratio aggregation methods. **(c)** Comparison of two

ratio aggregation methods in FragPipe based on protein noise-to-signal ratio (NSR) in the ccRCC whole proteome data. Box plots depict the NSR distributions in the NCI and QC samples, with p-values from t-tests labeled. The NSRs were calculated using 9322 proteins from five NCI samples and 9436 proteins from eight QC samples, and were compared against those from 182 patient samples, including 103 tumor and 79 normal samples. The box in each plot captures the IQR with the bottom and top edges representing the Q1 and Q3, respectively. The median (Q2) is indicated by a horizontal line within the box. The whiskers extend to the minima and maxima within 1.5 times the IQR below Q1 or above Q3. **(d)** Line charts showing the agreement between the median observed ratios and the theoretical ratios from both MS2 and MS3 data, with and without the "Best PSM" option enabled. **(e)** Same as (d), with and without the "Outlier removal" option enabled. Source data are provided as a Source Data file.

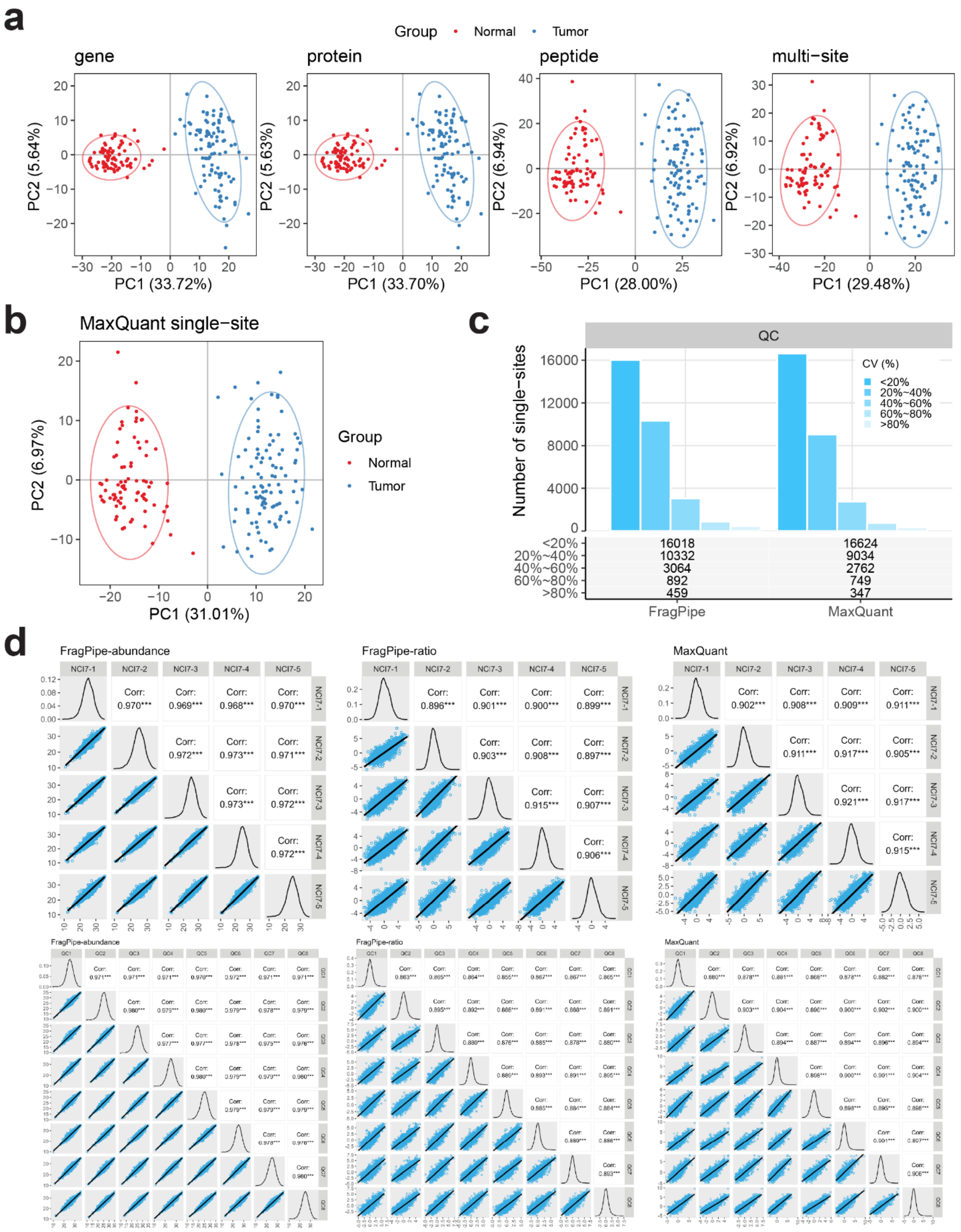

Supplementary Figure 4. Performance evaluations using the ccRCC phosphorylation-enriched dataset. (a) PCA plots of TMT-Integrator median-centered data at the gene, protein,

peptide, and multi-site levels from 103 tumor and 79 normal samples. **(b)** PCA plot of MaxQuant median-centered single-site data from 103 tumor and 79 normal samples. **(c)** Comparison of single-site CV distributions in QC samples. Bars represent the number of single sites in each CV group, with the counts listed at the bottom. **(d)** Evaluation of quantification consistency using FragPipe single-site abundance and ratio reports and the MaxQuant single-site ratio report for NCI and QC samples. Each subplot is labeled with the data type in the top left. The lower part shows linear fits, the diagonal displays density plots of abundances or ratios, and the upper part presents Pearson correlation test results, with correlation coefficients and significance levels (*) indicated. Source data are provided as a Source Data file.

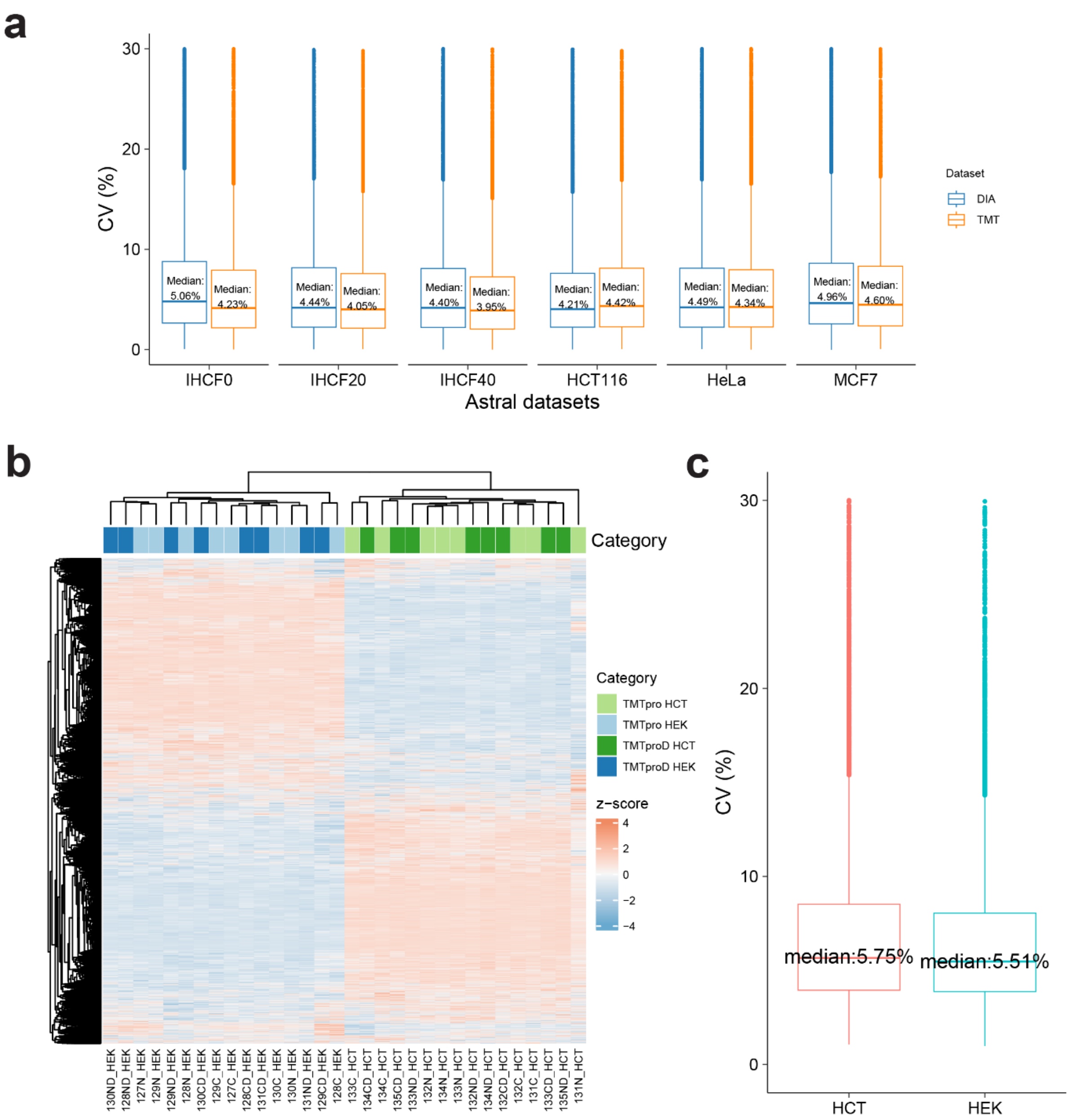

Supplementary Figure 5. Performance evaluations using the Astral and TMTpro 35-plex datasets. (a) Box plots comparing the CVs of the six cell types in the Astral TMT and DIA datasets. The box in each plot captures the IQR with the bottom and top edges representing the Q1 and Q3, respectively. The median (Q2) is indicated by a horizontal line within the box. The whiskers extend to the minima and maxima within 1.5 times the IQR below Q1 or above Q3. **(b)** Heatmap of unsupervised clustering of protein quantifications in the TMTpro 35-plex dataset. Rows represent proteins, columns represent channels, and the top annotation bar indicates cell type and reagent type. **(c)** Box plots showing CV distributions of each cell type in the TMTpro 35-plex dataset. The box in each plot captures the IQR with the bottom and top edges representing the Q1 and Q3, respectively. The median (Q2) is indicated by a horizontal line within the box. The

whiskers extend to the minima and maxima within 1.5 times the IQR below Q1 or above Q3. Source data are provided as a Source Data file.
