# Supplementary material for: Analysis of isobaric quantitative proteomic data using TMT-Integrator and FragPipe computational platform
Source: Nat Commun. 2026 Mar 2;17:4010. doi: 10.1038/s41467-026-70118-7 (PMC13136390; doi:10.1038/s41467-026-70118-7)
Supplement: Supplementary file 2 — Description of Additional Supplementary Files [file 41467_2026_70118_MOESM2_ESM.pdf]

Description of Additional Supplementary Files

File Name: Supplementary Data 1

Description: Spiked-in Protein Ratios.xlsx. The observed protein ratios calculated from the 12 spike-in proteins, used in Figure 4.

File Name: Supplementary Data 2

Description: FDP estimation.xlsx. The false discovery proportion (FDP) estimation results from entrapment database searches.

File Name: Supplementary Data 3

Description: OmicsEV final_evaluation_report.html. The OmicsEV evaluation report for the ccRCC whole-proteome dataset, used in Figure 3 and Table 1.
